# A Comparison of the Predictive Value of 12 Body Composition Markers for Metabolic Dysfunction-Associated Steatotic Liver Disease, At-Risk Metabolic Dysfunction-Associated Steatohepatitis, and Increased Liver Stiffness in a General Population Setting

**DOI:** 10.14309/ajg.0000000000003657

**Published:** 2025-07-23

**Authors:** Laurens A. van Kleef, Maurice Michel, Mesut Savas, Jesse Pustjens, Roel van de Laar, Edith Koehler, Elisabeth F.C. van Rossum, Harry L.A. Janssen, Jörn M. Schattenberg, Willem P. Brouwer

**Affiliations:** 1 Department of Gastroenterology and Hepatology, Erasmus MC, University Medical Center, Rotterdam, the Netherlands;; 2 Department of Internal Medicine, Ikazia Hospital, Rotterdam, the Netherlands;; 3 Department of Gastroenterology and Hepatology, Ikazia Hospital, Rotterdam, the Netherlands;; 4 Department of Internal Medicine II, Saarland University Medical Center, Homburg, Germany;; 5 Saarland University, Saarbrücken, Germany;; 6 Department of Internal Medicine, Division of Endocrinology, Erasmus MC, University Medical Center, Rotterdam, the Netherlands;; 7 Obesity Center CGG, Erasmus MC, University Medical Center, Rotterdam, the Netherlands;; 8 Toronto Centre for Liver Disease, Toronto General Hospital, University Health Network, Canada.

**Keywords:** MASLD, MASH, fibrosis, liver stiffness, general population, obesity, body composition, epidemiology

## Abstract

**INTRODUCTION:**

Adipose tissue is a key mediator of metabolic dysfunction-associated steatotic liver disease (MASLD) development and progression into metabolic dysfunction-associated steatohepatitis (MASH) and fibrosis. Since direct comparisons of body composition parameters are lacking, we here investigate 12 different body composition parameters.

**METHODS:**

Adult participants from National Health and Nutrition Examination Survey 2017–2023 with liver health data were included. Exclusion criteria were age older than 80 years, excessive alcohol (>60 g/d), viral hepatitis, and missing anthropometrics. MASLD was defined as controlled attenuation parameter ≥275 dB/m with metabolic dysfunction, MASH as FibroScan-aspartate aminotransferase ≥0.35, and increased liver stiffness measurement (LSM) as ≥8 kPa. Predictive performance of 12 body composition parameters was assessed using area under the curve analysis. Predicted probabilities of outcomes were visualized for standardized parameters, and nonlinearity was assessed through restricted cubic splines.

**RESULTS:**

Among 11,579 participants (age 51 [35–63], 47% male), 41% had MASLD, 6.5% at-risk MASH, and 9.9% increased LSM. Waist circumference (WC) and not BMI or waist-to-height ratio obtained the highest area under the curve for MASLD (0.82), at-risk MASH (0.73), and increased LSM (0.75) outperforming or equaling all other indices across subgroups. Associations between WC and MASLD were nonlinear, with slight risk saturation beyond 100 cm; at-risk MASH was linearly associated across the entire spectrum; and increased LSM risk rose only after WC >100 cm.

**DISCUSSION:**

In the general population, MASLD and MASH risk increased even when WC < 100 cm, while increased LSM risk was increasing only >100 cm. Although relatively minor differences, WC consistently demonstrated the highest predictive value for MASLD, at-risk MASH, and increased LSM and therefore most suited for MASLD diagnosis, management, and risk stratification.

## Graphical abstract

**Figure:**
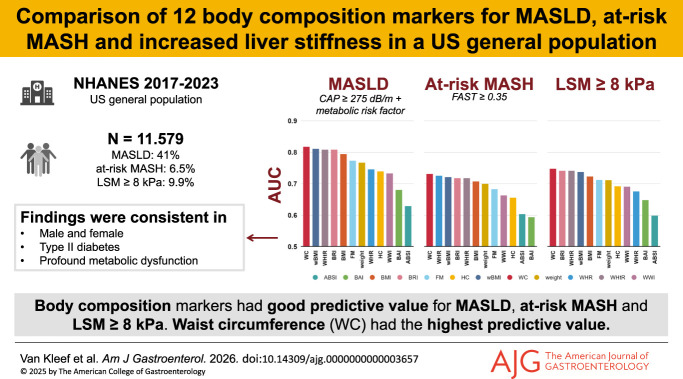

## INTRODUCTION

Metabolic dysfunction-associated steatotic liver disease (MASLD) prevalence has rapidly increased, currently affecting 1 in 3 individuals (1). This worrisome trend aligns with the surge in abdominal obesity and visceral fat deposition over the past few decades (2,3). Fat is an active endocrine organ which produces numerous hormones. Fat tissue plays a central role in regulating energy balance, metabolism, and immune function. However, when excessive fat mass accumulates, particularly in the abdominal region (visceral fat), the fat mass becomes dysfunctional and chronic inflammation occurs. This proinflammatory state is characterized by cytokine secretion, which is among others associated with dyslipidemia and insulin resistance (4–6). As a result, the visceral adipose tissue is a key mediator of MASLD development and progression into metabolic dysfunction-associated steatohepatitis (MASH) and fibrosis (7).

Traditionally, the body mass index (BMI) has been used to get an estimation of fat mass. However, BMI provides limited insight into fat distribution unlike other parameters that combine weight, height, waist circumference (WC), and/or hip circumference, e.g., body roundness index (BRI), waist-to-height ratio (WHtR), and waist-adjusted BMI (8). On the other hand, there are no standardized measurement sites for WC, and therefore, there is variability between readings, particularly in women (9,10). Nonetheless, the WC, WHtR, and waist-to-hip ratio have been adopted in the new framework for the diagnosis, staging, and management of obesity or new obesity definition (5,11). Moreover, algorithms such as a body shape index or calculated fat mass (FM) have been trained to estimate total FM and validated against dual-energy x-ray absorptiometry (12,13).

These body composition markers are associated with the risk of MASLD and adverse outcomes, due to the crucial role of visceral adipose tissue in liver health (14,15). However, a head-to-head comparison of these markers is lacking, leaving uncertainty about which body composition marker is most suited for MASLD diagnosis, management, and risk stratification.

In this population-based study, we directly compare 12 body composition parameters and the associations with MASLD, at-risk MASH, and increased liver stiffness measurements (LSM). Vibration controlled transient elastography (VCTE) with controlled attenuation parameter (CAP) and LSM was used as the reference standard in line with recent guidelines (16).

## METHODS

### Study population

This study was performed using National Health and Nutrition Examination Survey (NHANES) 2017–2023 study data. NHANES aimed to assess individuals' health and nutritional status of a US representative population. Data collection included extensive interviews, physical examination, clinical measurements, and tests by dedicated research assistants. Particularly relevant for this study were the anthropometric measurements including WC and hip circumference (HC), as well as the assessment of liver health with LSM and CAP. Further details on the aims, procedures, and design are available elsewhere (17,18). Participants with complete data on LSM and CAP were included. Exclusion criteria were age older than 80 years, daily alcohol consumption ≥60 g, viral hepatitis, and missing data on body composition parameters (weight, height, WC, and/or HC). Of note, viral hepatitis data were unavailable in NHANES 2021–2023 and could therefore not be excluded for. Data are publicly available from the NHANES database (https://www.cdc.gov/nchs/nhanes/index.htm).

### Body composition

Trained research assistants obtained WC measured at the iliac crest, HC, height, and weight which was either used directly as a parameter or used for the calculation of a body shape index, body adiposity index, BMI, BRI, FM, waist-to-hip ratio, WHtR, waist-adjusted BMI, and weight-adjusted waist index (12,19–23). All the formulas were based on body anthropometrics and are available in the Supplementary Digital Content (see Supplementary Table 2, http://links.lww.com/AJG/D717).

### Liver outcomes

VCTE was performed using the FibroScan model 502 V2 Touch (FibroScan, Echosens, Paris) in individuals who were requested to fast for at least 3 hours. There was access to both M-probe and XL-probe, which were used according to the device's instructions. LSM and CAP were simultaneously obtained and considered valid if at least 10 measurements with an interquartile range <30%. VCTE readings were subsequently used to define the following conditions:

1. MASLD: CAP ≥ 275 dB/m together with metabolic dysfunction (16,24).
2. At-risk MASH: FibroScan aspartate aminotransferase (AST) score ≥ 0.35 (25).
3. Increased LSM: LSM ≥ 8 kPa in the absence of heart failure (26,27).

Collectively, if any of these outcomes were present, it is referred to as “impaired liver health.”

### Covariates

Additional data used in this study were based on questionnaires (e.g., medical history for heart failure, presence of diabetes, hypertension, and use of medications) and blood samples (e.g., liver enzymes [only in NHANES 2017–2020], fasting glucose, HbA1c, high-density lipoprotein cholesterol). Metabolic dysfunction was assessed using the definitions outlined in the MASLD consensus statement and included overweight, (pre)diabetes, hypertension, high WC, and dyslipidemia (24).

### Statistical analysis

The predictive value of body composition parameters was evaluated using area under the curve (AUC) analysis. Differences in AUCs were tested for statistical significance using the DeLong test, with comparisons made between the best-performing test for each outcome and subgroup and the remaining tests. AUC analyses were conducted for the overall population, with subgroup analyses stratified by sex (male and female), diabetes status, and metabolic dysfunction (at least 2 criteria from the MASLD metabolic dysfunction definition).

Body composition parameters with skewed distributions were log transformed before analysis. All parameters were then standardized to a SD scale, also known as a Z-scale, by subtracting the mean followed by dividing the SD, to allow for a fair comparison. To assess the probability of outcomes on a continuous scale, plots were generated using locally estimated scatterplot smoothing.

Finally, nonlinearity was assessed by restricted cubic spline analysis using 3 knots among the standardized body composition parameters. The number of knots was further investigated by assessing the Akaike information criterion. Plots were created for WC, WHtR, BRI, and BMI to visualize the nonlinearity after adjusting for demographics and lifestyle (age, sex, ethnicity, smoking, and alcohol consumption). The risk was plotted using a reference of −1 SD because this population was overall metabolically unhealthy.

Analyses were performed in R version 4.0.4 (Foundation for Statistical Computing, Vienna, Austria). *P* values < 0.05 were considered statistically significant.

### Ethics

NHANES procedures and protocols were approved by the National Center for Health Statistics Research Ethics Review Board. Participants of the NHANES 2017–2020 cycle provided informed consent. This study was conducted according to the principles as outlined in the Declaration of Helsinki and Istanbul.

## RESULTS

### Participant disposition

The 2 independent NHANES cohorts comprised 12,967 adult participants with valid LSM and CAP measurements of whom 1,388 were excluded for either being of age older than 80 years, alcohol consumption ≥60 g/d, presence of viral hepatitis, or incomplete data on anthropometrics leaving 11,579 participants for analysis, see Supplementary Figure 1, http://links.lww.com/AJG/D717. The median age of participants was 51 [35–63] years: 47% were male, 19% had diabetes, and 67% had ≥ 2 metabolic dysfunction criteria (e.g., hypertension and abdominal obesity). MASLD was present in 4,731 of 11,579 (40.9%), at-risk MASH in 406 of 6,274 (6.5%), and LSM ≥ 8 kPa in 1,122 of 11,307 (9.9%). Additional baseline characteristics are provided in Table 1, and the distribution of the 12 investigated standardized body composition markers is given in Table 2.

**Table 1.**
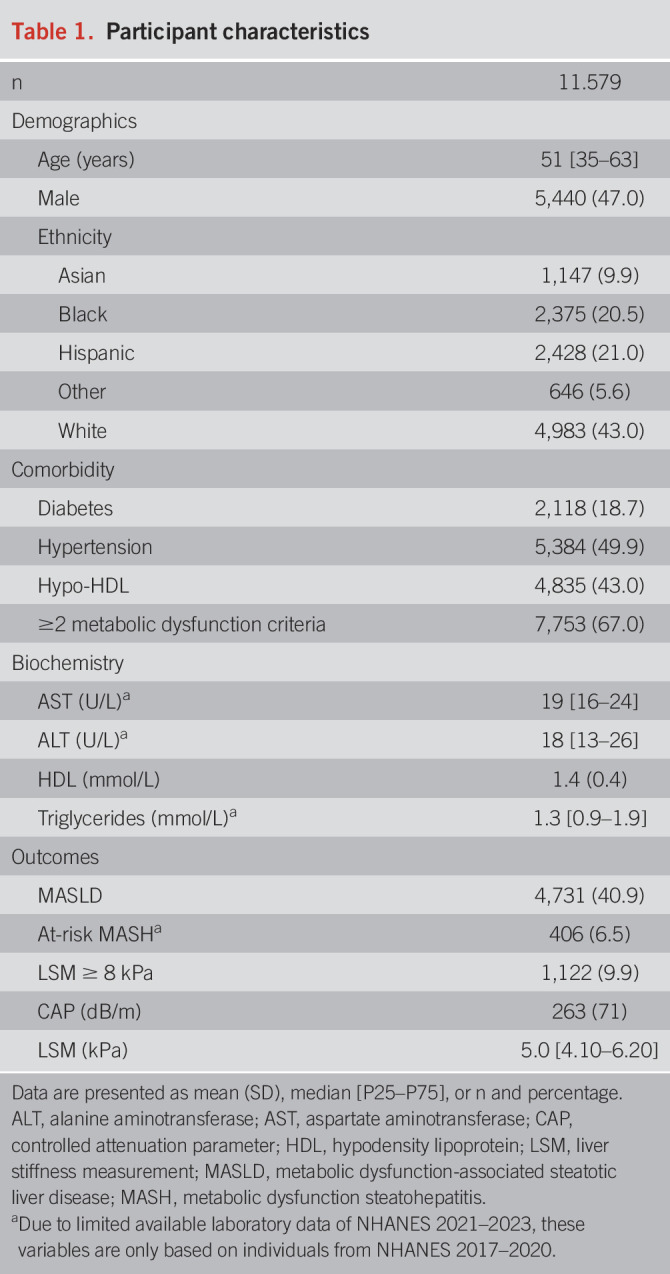
Participant characteristics

**Table 2.**
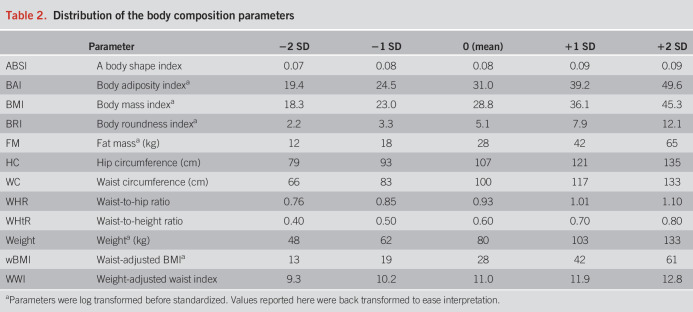
Distribution of the body composition parameters

### Body composition parameters in relation to MASLD, at-risk MASH, and increased LSM

**Figure 1.**
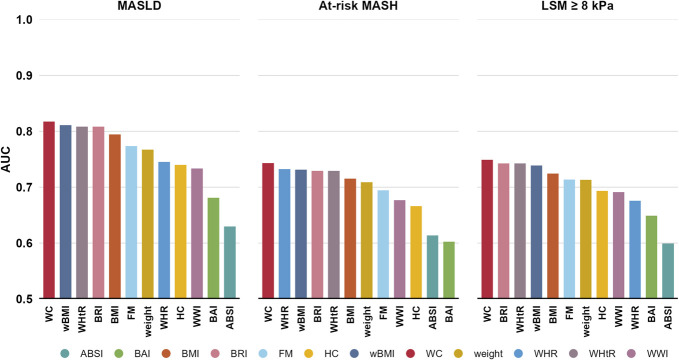
AUC per body composition marker for the detection of MASLD, at-risk MASH, and increased liver stiffness. MASLD was present in 4,731 of 11,579, at-risk MASH in 406 of 6,274, and LSM ≥ 8 kPa in 1,122 of 11,307 participants. AUC levels were compared among the body composition parameters using the DeLong test. *Indicates no significant difference with the best-performing test. ABSI, a body shape index; AUC, area under the curve; BAI, body adiposity index; BMI, body mass index; BRI, body roundness index; FM, fat mass; HC, hip circumference; LSM, liver stiffness measurement; MASLD, metabolic dysfunction-associated steatotic liver disease; MASH, metabolic dysfunction-associated steatohepatitis; wBMI, waist-adjusted BMI; WC, waist circumference; WHR, waist-hip ratio; WHtR, waist-height ratio; WWI, weight-adjusted waist index.

In the overall population, WC was the body composition parameter with the highest AUC on MASLD (AUC 0.82), at-risk MASH (AUC 0.73), and LSM ≥ 8 kPa (AUC 0.75), significantly outperforming all other investigated body composition parameters: Figure 1 and see Supplementary Table 1, http://links.lww.com/AJG/D717. Results were consistent in the NHANES 2017–2020 subgroup that had complete data on viral hepatitis status and when individuals with ALT > 100 IU/L were excluded.

### Body composition parameters and impaired liver health stratified by sex

**Figure 2.**
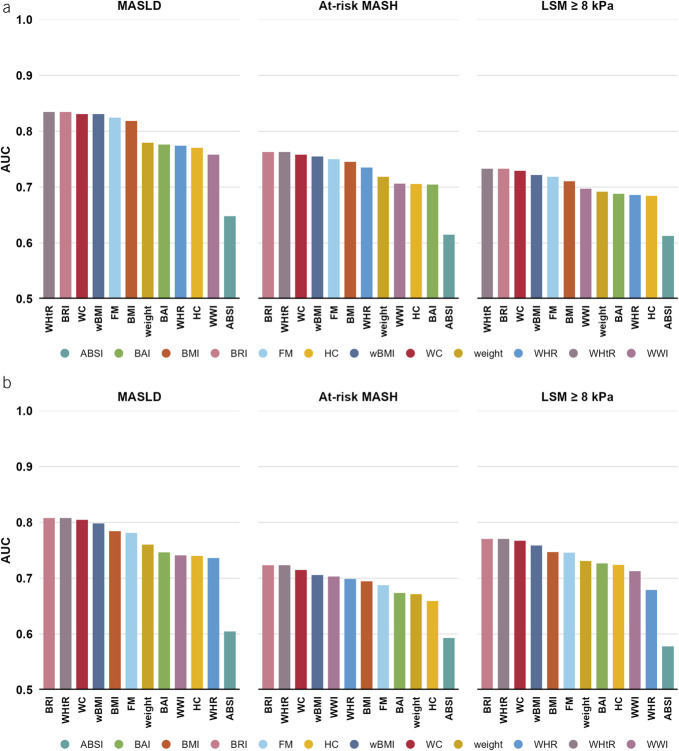
AUC per body composition marker for the detection of steatosis, at-risk MASH, and increased liver stiffness in male and female. In men, MASLD was present in 2,439 of 5,440, at-risk MASH in 264 of 3,020, and LSM ≥ 8 kPa in 597 of 5,277 participants. In women, MASLD was present in 2,292 of 6,139, at-risk MASH in 142 of 3,254, and LSM ≥ 8 kPa in 525 of 6,030 participants. AUC levels were compared among the body composition parameters using the DeLong test. *Indicates no significant difference with the best-performing test. ABSI, a body shape index; AUC, area under the curve; BAI, body adiposity index; BMI, body mass index; BRI, body roundness index; FM, fat mass; HC, hip circumference; LSM, liver stiffness measurement; MASLD, metabolic dysfunction-associated steatotic liver disease; MASH, metabolic dysfunction-associated steatohepatitis; wBMI, waist-adjusted BMI; WC, waist circumference; WHR, waist-hip ratio; WHtR, waist-height ratio; WWI, weight-adjusted waist index.

WHtR and BRI obtained the highest AUC in the sex-stratified analyses followed by WC for all investigated outcomes in both male and female, Figure 2. Body composition parameters were substantially more predictive for MASLD when compared with at-risk MASH and LSM ≥ 8 kPa. No large differences were observed between the predictive value of body composition parameters between men and women for impaired liver health. However, the best-performing body composition parameters had more predictive value for LSM ≥ 8 kPa in female (AUC of BRI and WHtR 0.77) compared with male (AUC BRI and WHtR 0.73), whereas there was more predictive value for at-risk MASH in male (AUC of BRI and WHtR 0.75) compared with female (AUC BRI and WHtR 0.71).

### Body composition parameters and impaired liver health by metabolic dysfunction

In at-risk subgroups with a higher prevalence of liver disease and higher body composition parameters, such as those with diabetes and those with ≥2 metabolic comorbidities, predictive values attenuated Supplementary Digital Content (see Supplementary Figure 2, http://links.lww.com/AJG/D717 and Supplementary Table 1, http://links.lww.com/AJG/D717). Still, WC was again among the tests with the highest predictive value for all outcomes in both subgroups.

### Impaired liver health by standardized body composition indices

**Figure 3.**
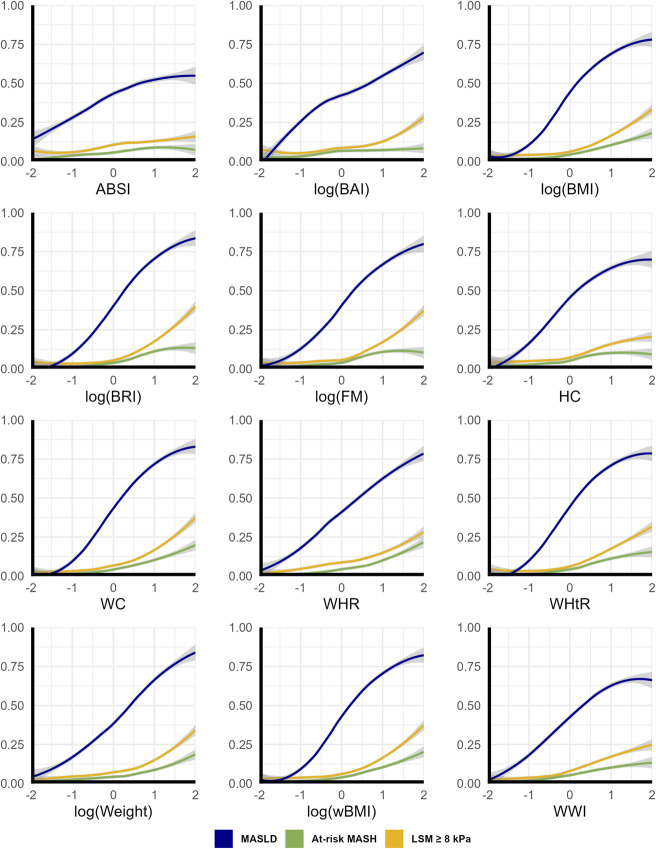
Predicted probability of MASLD, at-risk MASH, and increased LSM for standardized body composition parameters. Plots were generated using locally estimated scatterplot smoothing (LOESS) for all standardized body composition parameters. Table 2 can be used to correlate the standardized body composition parameters with values before transformation. ABSI, a body shape index; BAI, body adiposity index; BMI, body mass index; BRI, body roundness index; FM, fat mass; HC, hip circumference; LSM, liver stiffness measurement; MASLD, metabolic dysfunction-associated steatotic liver disease; MASH, metabolic dysfunction-associated steatohepatitis; wBMI, waist-adjusted BMI; WC, waist circumference; WHR, waist-hip ratio; WHtR, waist-height ratio; WWI, weight-adjusted waist index.

Next, the predicted probability of MASLD, at-risk MASH, and increased LSM for standardized body composition scores is presented in Figure 3. This indicates that the risk of MASLD and at-risk MASH increases across the entire body composition spectrum, even when the body composition marker is below the mean. This is in contrast with the predicted probability for increased LSM, which tends to only increase when the body composition parameter exceeds the mean.

### Predictive performance of body composition parameters for impaired liver health is nonlinear

**Figure 4.**
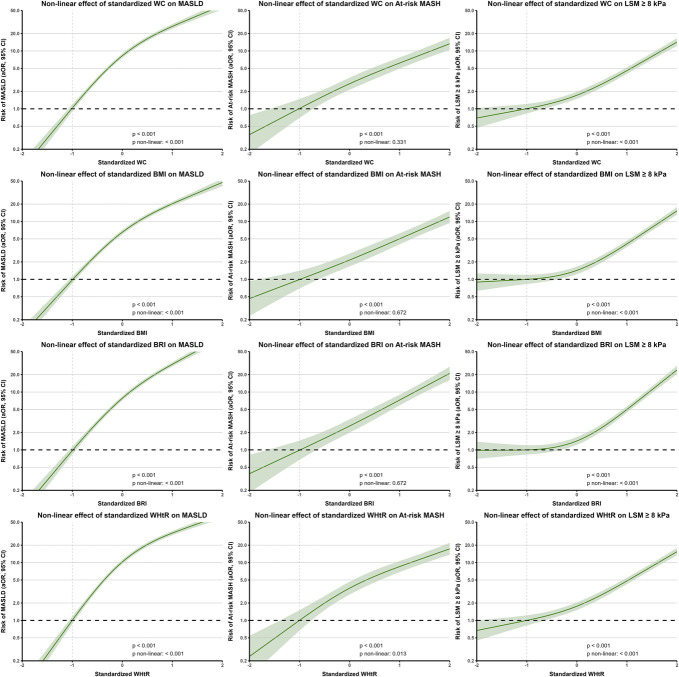
Nonlinear effect analysis of WC, BMI, BRI, and WHtR on MASLD, at-risk MASH, and increased liver stiffness using restricted cubic splines with 3 knots. Models were adjusted for age, sex, ethnicity, smoking, and alcohol consumption. Data on covariates were complete for 90.6% of participants resulting in MASLD being present in 4,368 of 10,486, at-risk MASH in 380 of 5,871, and LSM ≥ 8 kPa in 1,022 of 10,242 participants. Estimates were plotted against SD -1, corresponding to a body composition considered healthy (WC 83 cm, BMI 23 kg/m^2^, BRI 3.3, and WHtR 0.5, further correlation with Table 2). BMI, body mass index; BRI, body roundness index; LSM, liver stiffness measurement; MASLD, metabolic dysfunction-associated steatotic liver disease; MASH, metabolic dysfunction-associated steatohepatitis; WC, waist circumference; WHtR, waist-height ratio.

Finally, nonlinear effects were demonstrated for most body composition parameters in particular with MASLD and LSM ≥ 8 kPa and typically not with at-risk MASH (see Supplementary Table 2, http://links.lww.com/AJG/D717). The nonlinear effects, adjusted for age, sex, ethnicity, smoking, and alcohol consumption, are visualized in Figure 4 for the standardized body composition parameters with the most predictive value (WC, WHtR, and BRI) in previous analyses together with BMI. This analysis confirms that the risk of increased LSM only increases when the body composition parameter exceeds the mean, whereas the risk of MASLD and MASH tends to increase already from lower levels. In fact, the nonlinear association for MASLD with body composition parameters is only due to an attenuation of risk increase from mean + 0.5 SD and onward, indicating risk saturation. The adjusted predicted risk estimates of WHtR, BRI, WC, and BMI did not differ substantially.

## DISCUSSION

In this population-based, multiethnic cohort, we assessed the predictive value of 12 body composition parameters for impaired liver health. We demonstrated that WC had the most predictive value for all investigated outcomes: MASLD, at-risk MASH, and LSM ≥ 8 kPa (a surrogate of liver fibrosis). Although BRI and WHtR yielded numerically higher AUC levels in the sex-stratified analysis, WC was not statistically inferior for any of the outcomes in this population-based cohort of 11.579 individuals. Moreover, WC was the only body composition parameter that was consistently being among the parameters with the most predictive value in subgroups with diabetes or metabolic dysfunction. These findings support to include measurement of WC in MASLD management, particularly when BMI is not overtly elevated.

Visceral adipose tissue is a key factor in the onset, progression, and treatment response of MASLD and is therefore important to consider when managing MASLD (7,28,29). WC has previously been identified as an excellent marker for insulin resistance, which itself is a key driver of MASLD progression to MASH and fibrosis (30–32). In this line, in our study, WC also has the most predictive value for MASLD and its complications in a general population setting and outperformed BMI and other body composition parameters. Interestingly, it also outperformed a validated formula to estimate total FM (12). These findings align with cardiovascular study data summarized in a meta-analysis indicating that BMI was outperformed by WC and other indices (33). However, this meta-analysis indicated that WHtR might have even more predictive value, similar to our sex-stratified analysis. These findings once again underscore that the location of fat is important not only for cardiovascular risk but also for the risk of impaired liver health.

Although WC had more predictive value than BMI in all analyses, the overall difference in AUC is relatively small (AUC 0.72 vs 0.75). Transitioning from BMI to WC (although superior) might not change diagnosis nor management for most individuals, our findings still support including WC in assessing MASLD risk and managing the disease, in particular among individuals without overt obesity based on BMI. This aligns with 2 recent consensus statements that BMI alone is not sufficient to properly assess or manage cardiometabolic risk that is associated with increased adiposity. They conclude that WC needs to be fully incorporated in healthcare systems; even self-measured WC with instructions (e.g., https://myhealthywaist.org/my-waistline/) is a reliable option in settings where measuring WC is not feasible (11,34). These recommendations took into account that there is currently no standard measurement site for WC and it can be measured at the iliac crest as done in this study or at the level of the umbilicus. This might result in different measurements, particularly in female (10). Nonetheless regardless of the measurement site, WC had good accuracy for abdominal obesity (35).

Although one could argue that the predictive value of BMI is not that much different from WC, adopting WC might facilitate easier collaborations with other disciplines because it would allow for the calculation of the WHtR, the ratio between WC and height, an essential element of the framework for the diagnosis, staging, and management of obesity and new obesity definition (5,11). BRI does not have added value compared with WHtR because formulas are highly similar resulting in similar ranking. Complying with these new obesity frameworks is supported by this study from a liver health perspective and enables smooth collaboration with other healthcare professionals allowing for a holistic management approach (36).

Risk stratification is an important topic in the 2024 updated EASL-EASD-EASO clinical practice guideline on MASLD and a research priority (28,37). Including WC or WC-based indices in risk stratification algorithms may be a step in the right direction, especially for a general population setting. Indeed, a recent comparison of 10 NITs in a general population setting, the metabolic dysfunction associated fibrosis (MAF-5) score that included WC had the most predictive value for impaired liver health (e.g., *increased LSM, at-risk MASH or cirrhosis*). It has also been shown that the Fibrosis-4 (FIB-4) has a less predictive value in a population-based setting (typically AUC < 0.6 for LSM ≥ 8 kPa) than WC and body composition-based algorithms such as the steatosis associated fibrosis estimator (SAFE) and MAF-5 score (15,38–40). With current noninvasive tests, even small increases in AUC levels might have important consequences for the feasibility of referral strategies and the workload in already strained healthcare systems. In addition, more accurate risk stratification could help in the more targeted identification of eligible study candidates for ongoing trials, which is currently particularly difficult (41).

Another important finding of this study is the nonlinearity of fibrosis risk, as illustrated by a stable risk of increased LSM until the mean, which then rapidly increases with increasing body composition parameter levels, which persisted after adjusting for demographics and metabolic health. This stable risk for LSM ≥ 8 kPa until the mean in this study was in contrast with the risk of MASLD and at-risk MASH, which increases across the entire spectrum of the body composition parameters but for MASLD tends to saturate at higher levels. Insulin resistance may play a crucial role in these findings because insulin resistance is recognized as a strong predictor of changes in impaired liver health (32). Interestingly, the threshold for which LSM ≥ 8 kPa risk started to increase more rapidly in this study was a WC of 100 cm, which was identified as a cutoff to rule out insulin resistance in men and women with an negative predictive value (NPV) of 0.98 and 0.97, respectively, while maintaining a sensitivity of 94%–98% (30). This is a remarkable finding, indicating that fattening and inflammation of the liver occur along the entire spectrum of abdominal fat gain, whereas scarring of the liver tends to only substantially increase when obesity is poorly managed.

Finally, the nonlinear risk for increased LSM indicates that noninvasive tests may benefit from truncating the body composition parameter at the lower level. These findings also highlight a window of opportunity by addressing obesity early to reduce the onset of obesity-related hepatic injury. Furthermore, our results suggest that intervening in overweight individuals may already help mitigate fat accumulation in the liver, highlighting the need for lifestyle interventions before obesity develops.

### Limitations

Although this study has a large sample size and comprehensively assessed the predictive value of 12 body composition parameters regarding MASLD, at-risk MASH, and increased liver stiffness, the following limitations should be taken into account. First, this was a cross-sectional study, and no data were available on the trajectories of body composition or liver outcomes. Second, we were unable to calculate the FibroScan AST score because of missing data on AST in NHANES 2021–2023, and the reported at-risk MASH analyses were thus performed in the NHANES 2017–2020 subset only. In continuation of this, no exclusions for viral hepatitis could be performed in NHANES 2021–2023, which in NHANES 2017–2020 was rare (<1%). However, the outcomes for the subset which had complete data on exclusion criteria were consistent with the outcomes in the overall population. Third, LSM might be unreliable in individuals with elevated ALT levels. However, the results were consistent in a subset of NHANES where the 50 individuals with ALT >100 IU/L were excluded (remaining n = 11,529; data not shown). Fourth, there is an ongoing debate on whether obesity itself falsely elevates liver stiffness and thereby explains the strong correlation between body composition parameters and liver outcomes (42,43). However, a recent meta-analysis among 10.537 patients indicated that LSM was reliable in individuals with a BMI > 30 kg/m^2^ when using the XL-probe, unlike CAP levels, which might be falsely elevated even when using the XL-probe (44). Although biopsy data to confirm these findings would be very interesting, obtaining these in a general population setting is considered unethical; hence, we depend on surrogates such as vibration-controlled transient elastography. Fifth, no stratification was made for alcohol consumption because of the groups being too small to actually report the thresholds for MetALD in line with previous analysis in NHANES (45). Finally, this study did not compare body composition parameters with NITs extensively and due to a lack of data on common parameters such as ALT and AST in NHANES after 2021.

### Conclusion

Body composition parameters had a high predictive value for impaired liver health, with WC being the body composition parameter with the highest predictive value for MASLD, at-risk MASH, and increased LSM in a general population setting and in individuals with more profound metabolic dysfunction: diabetes or the presence of multiple metabolic dysfunction criteria. WC-based body composition markers such as BRI or WHtR did not have a significantly additional predictive value compared with WC alone. WC, particularly in the absence of overt obesity, should be considered as the preferred body composition parameter in individuals at risk of MASLD, MASH, or fibrosis.

## CONFLICTS OF INTEREST

**Guarantor of the article:** Willem P. Brouwer, MD, PhD.

**Specific author contributions:** L.v.K., W.P.B.: collection of data. L.v.K., M.S., M.M., J.M.S., W.P.B.: study design, data analysis, writing of the manuscript. Critical review of the manuscript, writing of the manuscript, approval of final version, and approval of submission: all authors.

**Financial support:** Financial support was provided by the Foundation for Liver and Gastrointestinal Research, Rotterdam, the Netherlands. The funding source did not influence the study design, data collection, analysis and interpretation of the data, nor the writing of the report and decision to submit for publication.

**Potential competing interests:** M.M. Speaker honorarium from Ipsen, NovoNordisk, and Gilead Sciences. J.M.S. declares consultant honorary from Akero, Alentis, Alexion, Altimmune, Astra Zeneca, 89Bio, Bionorica, Boehringer Ingelheim, Gilead Sciences, GSK, HistoIndex, Ipsen, Inventiva Pharma, Madrigal Pharmaceuticals, Kríya Therapeutics, Lilly, MSD Sharp & Dohme GmbH, Nordic Bioscience, Northsea Therapeutics, Novartis, Novo Nordisk, Pfizer, Roche, Sanofi, Siemens Healthineers, Summit Clinical, and Vantage Biosciences Research; speaker honorarium from AbbVie, Boehringer Ingelheim, Gilead Sciences, Ipsen Novo Nordisk, Madrigal Pharmaceuticals, and Worldwide Clinical Trials, Stockholder options: Hepta Bio. H.L.A.J. received grants from AbbVie, Arbutus, Gilead Sciences, Janssen, and Roche, and is a consultant for Arbutus, Arena, Enyo, Gilead Sciences, GlaxoSmithKline (GSK), Janssen, Merck, Roche, Vir Biotechnology Inc., and Viroclinics. E.F.C.v.R. has previously been involved in the medical care for patient participating in a clinical trial from Rhythm Pharmaceuticals, Inc (no personal funding) and received personal royalties for the lay book FAT the secret organ. W.P.B. received speakers fees for Eli Lilly, is part of the advisory board of Novo Nordisk and participates in trials of 89BIO, Boehringer Ingelheim, Novo Nordisk, and Inventiva Pharma. The other authors had no conflicts of interest with respect to the current work.

**Data transparency statement:** Data are publicly available from the NHANES database (https://www.cdc.gov/nchs/nhanes/index.htm).

**Study Highlights**

**WHAT IS KNOWN**

✓ Adipose tissue is a key factor in metabolic dysfunction-associated steatotic liver disease (MASLD) development and progression into advanced liver disease.
✓ BMI alone is not sufficient to fully characterize adipose tissue.
✓ There is uncertainty about which body composition marker is most suited for MASLD diagnosis, management, and risk stratification.

**WHAT IS NEW HERE**

✓ Waist circumference had the most predictive value for MASLD, at-risk metabolic dysfunction-associated steatohepatitis, and increased liver stiffness, significantly outperforming the body mass index.
✓ Waist circumference-based indices such as waist-to-height ratio or waist-to-hip ratio did not yield more predictive value.
✓ The risk of MASLD and at-risk metabolic dysfunction-associated steatohepatitis increased across the entire body composition spectrum, while increased liver stiffness measurement risk rose only after exceeding waist circumference > 100 cm.
✓ WC could have additional value in MASLD diagnosis, management, and risk stratification.

## Supplementary Material

## ABBREVIATIONS:

ABSI: a body shape index
AIC: Akaike information criterion
ALT: alanine aminotransferase
AST: aspartate aminotransferase
AUC: area under the curve
BAI: body adiposity index
BMI: body mass index
BRI: body roundness index
CAP: controlled attenuation parameter
FM: fat mass
HC: hip circumference
HbA1c: glycated hemoglobin
HDL-c: high-density lipoprotein cholesterol
kPa: kilopascal
LSM: liver stiffness measurement
MASH: metabolic dysfunction-associated steatohepatitis
MASLD: metabolic dysfunction-associated steatotic liver disease
NHANES: National Health and Nutrition Examination Survey
wBMI: waist-adjusted body mass index
WC: waist circumference
WHR: waist-to-hip ratio
WHtR: waist-to-height ratio
WWI: weight-adjusted waist index

## References

[1] Le MH Yeo YH Li X 2019 global NAFLD prevalence: A systematic review and meta-analysis. Clin Gastroenterol Hepatol 2022;20(12):2809–17.e28. 34890795 10.1016/j.cgh.2021.12.002

[2] NCD Risk Factor Collaboration NCD-RisC. Worldwide trends in underweight and obesity from 1990 to 2022: A pooled analysis of 3663 population-representative studies with 222 million children, adolescents, and adults. Lancet 2024;403(10431):1027–50. 38432237 10.1016/S0140-6736(23)02750-2 PMC7615769

[3] Boutari C Mantzoros CS. A 2022 update on the epidemiology of obesity and a call to action: As its twin COVID-19 pandemic appears to be receding, the obesity and dysmetabolism pandemic continues to rage on. Metabolism 2022;133:155217. 35584732 10.1016/j.metabol.2022.155217 PMC9107388

[4] Jung UJ Choi M-S. Obesity and its metabolic complications: The role of adipokines and the relationship between obesity, inflammation, insulin resistance, dyslipidemia and nonalcoholic fatty liver disease. Int J Mol Sci 2014;15(4):6184–223. 24733068 10.3390/ijms15046184 PMC4013623

[5] Busetto L Dicker D Frühbeck G . A new framework for the diagnosis, staging and management of obesity in adults. Nat Med 2024;30(9):2395–9. 38969880 10.1038/s41591-024-03095-3

[6] Yumuk V Tsigos C Fried M . European guidelines for obesity management in adults. Obes Facts 2015;8(6):402–24. 26641646 10.1159/000442721 PMC5644856

[7] Bansal S Vachher M Arora T . Visceral fat: A key mediator of NAFLD development and progression. Hum Nutr Metab 2023;33:200210.

[8] Madden AM Smith S. Body composition and morphological assessment of nutritional status in adults: A review of anthropometric variables. J Hum Nutr Diet 2016;29(1):7–25. 10.1111/jhn.12278 25420774

[9] Wang J Thornton JC Bari S . Comparisons of waist circumferences measured at 4 sites. Am J Clin Nutr 2003;77(2):379–84. 12540397 10.1093/ajcn/77.2.379

[10] Mason C Katzmarzyk PT. Variability in waist circumference measurements according to anatomic measurement site. Obesity (Silver Spring) 2009;17(9):1789–95. 19343017 10.1038/oby.2009.87

[11] Rubino F Cummings DE Eckel RH . Definition and diagnostic criteria of clinical obesity. Lancet Diabetes Endocrinol 2025;13(3):221–62. 39824205 10.1016/S2213-8587(24)00316-4 PMC11870235

[12] Lee DH Keum N Hu FB . Development and validation of anthropometric prediction equations for lean body mass, fat mass and percent fat in adults using the national Health and Nutrition Examination Survey (NHANES) 1999–2006. Br J Nutr 2017;118(10):858–66. 29110742 10.1017/S0007114517002665

[13] Gómez-Ambrosi J Silva C Catalán V . Clinical usefulness of a new equation for estimating body fat. Diabetes Care 2012;35(2):383–8. 22179957 10.2337/dc11-1334 PMC3263863

[14] Priego-Parra BA Reyes-Diaz SA Ordaz-Alvarez HR . Diagnostic performance of sixteen biomarkers for MASLD: A study in a Mexican cohort. Clin Res Hepatol Gastroenterol 2024;48(7):102400. 38901566 10.1016/j.clinre.2024.102400

[15] Graupera I Thiele M Serra-Burriel M . Low accuracy of FIB-4 and NAFLD fibrosis scores for screening for liver fibrosis in the population. Clin Gastroenterol Hepatol 2022;20(11):2567–76.e6. 34971806 10.1016/j.cgh.2021.12.034

[16] European Association for the Study of the Liver. EASL Clinical Practice Guidelines on non-invasive tests for evaluation of liver disease severity and prognosis—2021 update. J Hepatol 2021;75(3):659–89. 34166721 10.1016/j.jhep.2021.05.025

[17] Johnson CL Paulose-Ram R Ogden CL . National health and nutrition examination survey. Analytic guidelines, 1999-2010. Vital Health Stat 2 2013;161:1–24. 25090154

[18] Akinbami LJ Chen T-C Davy O . National health and nutrition examination Survey, 2017-March 2020 prepandemic file: Sample Design, Estimation, and Analytic Guidelines. Vital Health Stat 1 2022(190):1–36. 35593699

[19] Thomas DM Bredlau C Bosy-Westphal A . Relationships between body roundness with body fat and visceral adipose tissue emerging from a new geometrical model. Obesity 2013;21(11):2264–71. 23519954 10.1002/oby.20408 PMC3692604

[20] Krakauer NY Krakauer JC. A new body shape index predicts mortality hazard independently of body mass index. PLoS One 2012;7:e39504. 22815707 10.1371/journal.pone.0039504 PMC3399847

[21] Bergman RN Stefanovski D Buchanan TA . A better index of body adiposity. Obesity (Silver Spring) 2011;19(5):1083–9. 21372804 10.1038/oby.2011.38 PMC3275633

[22] Antonini-Canterin F Di Nora C Poli S . Obesity, Cardiac remodeling, and metabolic profile: Validation of a new simple Index beyond body mass index. J Cardiovasc Echogr 2018;28(1):18–25. 29629255 10.4103/jcecho.jcecho_63_17 PMC5875131

[23] Park Y Kim NH Kwon TY . A novel adiposity index as an integrated predictor of cardiometabolic disease morbidity and mortality. Scientific Rep 2018;8(1):16753. 10.1038/s41598-018-35073-4 PMC6233180 30425288

[24] Rinella ME Lazarus JV Ratziu V . A multisociety Delphi consensus statement on new fatty liver disease nomenclature. J Hepatol 2023;79(6):1542–56. 37364790 10.1016/j.jhep.2023.06.003

[25] Newsome PN Sasso M Deeks JJ . FibroScan-AST (FAST) score for the non-invasive identification of patients with non-alcoholic steatohepatitis with significant activity and fibrosis: A prospective derivation and global validation study. Lancet Gastroenterol Hepatol 2020;5(4):362–73. 32027858 10.1016/S2468-1253(19)30383-8 PMC7066580

[26] Roulot D Costes JL Buyck JF . Transient elastography as a screening tool for liver fibrosis and cirrhosis in a community-based population aged over 45 years. Gut 2011;60(7):977–84. 21068129 10.1136/gut.2010.221382

[27] van Kleef LA Sonneveld MJ Zhu F . Liver stiffness is associated with excess mortality in the general population driven by heart failure: The Rotterdam study. Liver Int 2023;43(5):1000–7. 36744819 10.1111/liv.15538

[28] European Association for the Study of the Liver EASLEuropean Association for the Study of Diabetes EASDEuropean Association for the Study of Obesity EASO. EASL–EASD–EASO clinical Practice Guidelines on the management of metabolic dysfunction-associated steatotic liver disease (MASLD). J Hepatol 2024;81(3):492–542. 38851997 10.1016/j.jhep.2024.04.031

[29] Shen W Middleton MS Cunha GM . Changes in abdominal adipose tissue depots assessed by MRI correlate with hepatic histologic improvement in non-alcoholic steatohepatitis. J Hepatol 2023;78(2):238–46. 36368598 10.1016/j.jhep.2022.10.027 PMC9852022

[30] Wahrenberg H Hertel K Leijonhufvud B-M . Use of waist circumference to predict insulin resistance: Retrospective study. BMJ 2005;330(7504):1363–4. 15833749 10.1136/bmj.38429.473310.AE PMC558285

[31] Israelsen M Francque S Tsochatzis EA . Steatotic liver disease. Lancet 2024;404(10464):1761–78. 39488409 10.1016/S0140-6736(24)01811-7

[32] Wernberg CW Chandran VI Lauridsen MM . Ability of soluble TREM2 and PRO-C3 as biomarkers to predict changes in MASLD activity. JHEP Rep 2025;7(8):101432. 40677693 10.1016/j.jhepr.2025.101432 PMC12269582

[33] Ashwell M Gunn P Gibson S. Waist-to-height ratio is a better screening tool than waist circumference and BMI for adult cardiometabolic risk factors: Systematic review and meta-analysis. Obes Rev 2012;13(3):275–86. 22106927 10.1111/j.1467-789X.2011.00952.x

[34] Ross R Neeland IJ Yamashita S . Waist circumference as a vital sign in clinical practice: A consensus statement from the IAS and ICCR Working Group on Visceral Obesity. Nat Rev Endocrinol 2020;16(3):177–89. 32020062 10.1038/s41574-019-0310-7 PMC7027970

[35] Ostchega Y Seu R Isfahani NS Waist circumference measurement methodology study: National Health and Nutrition Examination Survey, 2016. Vital Health Stat 2 2019;182:1–20. 30707674

[36] Zeng J Fan J-G Francque SM. Therapeutic management of metabolic dysfunction associated steatotic liver disease. United Eur Gastroenterol J 2024;12(2):177–86. 10.1002/ueg2.12525 PMC10954426 38193865

[37] Lazarus JV Mark HE Allen AM . A global research priority agenda to advance public health responses to fatty liver disease. J Hepatol 2023;79(3):618–34. 37353401 10.1016/j.jhep.2023.04.035

[38] van Kleef LA Francque SM Prieto-Ortiz JE . Metabolic dysfunction-associated fibrosis 5 (MAF-5) score predicts liver fibrosis risk and outcome in the general population with metabolic dysfunction. Gastroenterology 2024;167(2):357–67.e9. 38513745 10.1053/j.gastro.2024.03.017

[39] Sripongpun P Kim WR Mannalithara A . The steatosis-associated fibrosis estimator (SAFE) score: A tool to detect low-risk NAFLD in primary care. Hepatology 2023;77(1):256–67. 35477908 10.1002/hep.32545 PMC9613815

[40] van Kleef LA Pustjens J Schattenberg JM, et al. Comparison of diagnostic accuracy and utility of non-invasive tests for clinically significant liver disease in a general population with metabolic dysfunction. Hepatology. 2025. doi:10.1097/HEP.0000000000001356. . In press. 40331893

[41] Ratziu V Harrison SA Hajji Y . NIS2+^TM^ as a screening tool to optimize patient selection in metabolic dysfunction-associated steatohepatitis clinical trials. J Hepatol 2024;80(2):209–19. 38061448 10.1016/j.jhep.2023.10.038

[42] Wong GLH Chan HLY Choi PCL . Association between anthropometric parameters and measurements of liver stiffness by transient elastography. Clin Gastroenterol Hepatol 2013;11(3):295–302.e3023. 23022698 10.1016/j.cgh.2012.09.025

[43] Wai JW Fu C Wong VW-S. Confounding factors of non-invasive tests for nonalcoholic fatty liver disease. J Gastroenterol 2020;55(8):731–41. 32451628 10.1007/s00535-020-01686-8 PMC7376510

[44] Cao Y-t Xiang L-l Qi F . Accuracy of controlled attenuation parameter (CAP) and liver stiffness measurement (LSM) for assessing steatosis and fibrosis in non-alcoholic fatty liver disease: A systematic review and meta-analysis. eClinicalMedicine 2022;51:101547. 35844772 10.1016/j.eclinm.2022.101547 PMC9284399

[45] Kim D Loomba R Ahmed A. Current burden of MASLD, MetALD, and hepatic fibrosis among US adults with prediabetes and diabetes, 2017-2023. Clin Mol Hepatol 2025;31(3):e235–8. 39736264 10.3350/cmh.2024.1150 PMC12260613

