# Supplementary material for: A Comparison of the Predictive Value of 12 Body Composition Markers for Metabolic Dysfunction-Associated Steatotic Liver Disease, At-Risk Metabolic Dysfunction-Associated Steatohepatitis, and Increased Liver Stiffness in a General Population Setting
Source: Am J Gastroenterol. 2025 Jul 23;121(6):1382–92. doi: 10.14309/ajg.0000000000003657 (PMC13225109; doi:10.14309/ajg.0000000000003657)
Supplement: Supplementary file 1 [file acg-121-1382-s001.docx]

**A comparison of the predictive value of 12 body composition markers for MASLD, at-risk MASH and increased liver stiffness in a general population setting**

Laurens A. van Kleef^1,2,3^, Maurice Michel^4,5^, Mesut Savas^6,7^, Jesse Pustjens^1^, Roel van de Laar^2^, Edith Koehler^3^, Elisabeth F.C. van Rossum^6,7^, Harry L.A. Janssen^1,8^, Jörn M. Schattenberg^4,5^ and Willem P. Brouwer^1^

1. Department of Gastroenterology and Hepatology, Erasmus MC, University Medical Center, Rotterdam, The Netherlands

2. Department of Internal Medicine, Ikazia Hospital, Rotterdam, The Netherlands

3. Department of Gastroenterology and Hepatology, Ikazia Hospital, Rotterdam, The Netherlands

4. Department of Internal Medicine II, Saarland University Medical Center, Homburg, Germany

5. Saarland University, Saarbrücken, Germany

6. Department of Internal Medicine, Division of Endocrinology, Erasmus MC, University Medical Center, Rotterdam, The Netherlands

7. Obesity Center CGG, Erasmus MC, University Medical Center, Rotterdam, The Netherlands

8. Toronto Centre for Liver Disease, Toronto General Hospital, University Health Network, Canada

**SUPPLEMENTARY FILES**

| **Supplementary table 1: AUC level for the prediction of MASLD, at-risk MASH and increased liver stiffness for the investigated body composition parameters in all groups.** | | | | |
| --- | --- | --- | --- | --- |
|  |  | **MASLD** | **At-risk MASH** | **LSM ≥ 8 kPa** |
| **All** | **ABSI** | 0.63 | 0.60 | 0.60 |
|  | **BAI** | 0.68 | 0.59 | 0.65 |
|  | **BMI** | 0.79 | 0.71 | 0.72 |
|  | **BRI** | 0.81 | 0.72 | 0.74 |
|  | **FM** | 0.77 | 0.68 | 0.71 |
|  | **HC** | 0.74 | 0.66 | 0.69 |
|  | **wBMI** | 0.81 | 0.72 | 0.74 |
|  | **WC** | **0.82** | **0.73** | **0.75** |
|  | **weight** | 0.77 | 0.70 | 0.71 |
|  | **WHR** | 0.75 | 0.73 | 0.68 |
|  | **WHtR** | 0.81 | 0.72 | 0.74 |
|  | **WWI** | 0.73 | 0.66 | 0.69 |
| **Male** | **ABSI** | 0.65 | 0.60 | 0.61 |
|  | **BAI** | 0.78 | 0.70 | 0.69 |
|  | **BMI** | 0.82 | 0.74 | 0.71 |
|  | **BRI** | **0.83** | **0.75** | **0.73** |
|  | **FM** | 0.82 | 0.74 | 0.72 |
|  | **HC** | 0.77 | 0.70 | 0.68 |
|  | **wBMI** | 0.83 | 0.75 | 0.72 |
|  | **WC** | 0.83 | 0.75 | 0.73 |
|  | **weight** | 0.78 | 0.71 | 0.69 |
|  | **WHR** | 0.77 | 0.72 | 0.69 |
|  | **WHtR** | **0.83** | **0.75** | **0.73** |
|  | **WWI** | 0.76 | 0.69 | 0.70 |
| **Female** | **ABSI** | 0.60 | 0.59 | 0.58 |
|  | **BAI** | 0.75 | 0.66 | 0.73 |
|  | **BMI** | 0.78 | 0.68 | 0.75 |
|  | **BRI** | **0.81** | **0.71** | **0.77** |
|  | **FM** | 0.78 | 0.67 | 0.74 |
|  | **HC** | 0.74 | 0.65 | 0.72 |
|  | **wBMI** | 0.80 | 0.69 | 0.76 |
|  | **WC** | 0.80 | 0.70 | 0.77 |
|  | **weight** | 0.76 | 0.66 | 0.73 |
|  | **WHR** | 0.74 | 0.69 | 0.68 |
|  | **WHtR** | **0.81** | **0.71** | **0.77** |
|  | **WWI** | 0.74 | 0.69 | 0.71 |
| **Diabetes** | **ABSI** | 0.51 | 0.52 | 0.50 |
|  | **BAI** | 0.65 | 0.56 | 0.63 |
|  | **BMI** | 0.74 | 0.66 | 0.70 |
|  | **BRI** | 0.73 | 0.65 | 0.70 |
|  | **FM** | 0.72 | 0.63 | 0.69 |
|  | **HC** | 0.70 | 0.61 | 0.68 |
|  | **wBMI** | **0.74** | 0.66 | **0.71** |
|  | **WC** | 0.74 | **0.66** | 0.71 |
|  | **weight** | 0.72 | 0.65 | 0.69 |
|  | **WHR** | 0.62 | 0.63 | 0.58 |
|  | **WHtR** | 0.73 | 0.65 | 0.70 |
|  | **WWI** | 0.63 | 0.58 | 0.61 |
| **Metabolic Dysfunction** | **ABSI** | 0.55 | 0.55 | 0.55 |
|  | **BAI** | 0.61 | 0.53 | 0.62 |
|  | **BMI** | 0.73 | 0.66 | 0.70 |
|  | **BRI** | 0.74 | 0.66 | 0.72 |
|  | **FM** | 0.70 | 0.63 | 0.69 |
|  | **HC** | 0.68 | 0.60 | 0.67 |
|  | **wBMI** | 0.75 | 0.67 | 0.72 |
|  | **WC** | **0.75** | 0.68 | **0.73** |
|  | **weight** | 0.71 | 0.66 | 0.69 |
|  | **WHR** | 0.67 | **0.69** | 0.63 |
|  | **WHtR** | 0.74 | 0.66 | 0.72 |
|  | **WWI** | 0.64 | 0.60 | 0.65 |
| Numbers in bold indicate the numerically highest AUC level for the specific outcome and specific subgroup. Underlined AUC levels were not significantly different from the body composition parameter with the highest predictive value.  Abbreviations: ABSI, a body shape index; BAI, body adiposity index; BMI, body mass index; BRI, body roundness index; FM, fat mass; HC, hip circumference; LSM, liver stiffness measurement; MASLD, metabolic dysfunction associated steatotic liver disease; MASH, metabolic dysfunction associated steatohepatitis; wBMI, waist adjusted BMI; WC, waist circumference; WHR, Waist Hip ratio; WHtR, Waist Height Ratio; WWI, weight adjusted waist index. | | | | |

| **Supplementary table 2: detailed characteristics of the body composition parameters used in this study** | | |
| --- | --- | --- |
|  | **Parameter** | **Formula** |
| ABSI | A Body Shape Index | WC / (BMI^2/3^ * height^1/2^) |
| BAI | Body Adiposity Index | (HC / height^1.5^) - 18 |
| BMI | Body Mass Index | weight / (height^2^) |
| BRI | Body Roundness Index | 364.2 - 365.5 × {1 − [(WC/2π)/(0.5 × height)]^2^}^0.5^ |
| FM | Fat Mass | Male: -18.592 - age * 0.009 - height * 0.080 + weight * 0.226 + WC * 0.387  Female: 11.817 + age * 0.041 - height * 0.199 + weight * 0.610 + WC * 0.044 |
| HC | Hip Circumference | - |
| WC | Waist Circumference | - |
| WHR | Waist-to-Hip Ratio | WC / HC |
| WHtR | Waist-to-Height Ratio | WC / height |
| Weight | Weight | - |
| wBMI | Waist adjusted BMI | WC * BMI |
| WWI | Weight adjusted waist index | WC / weight^1/2^ |
|  | | |

**Supplementary Figure 1: Study flowchart**

**
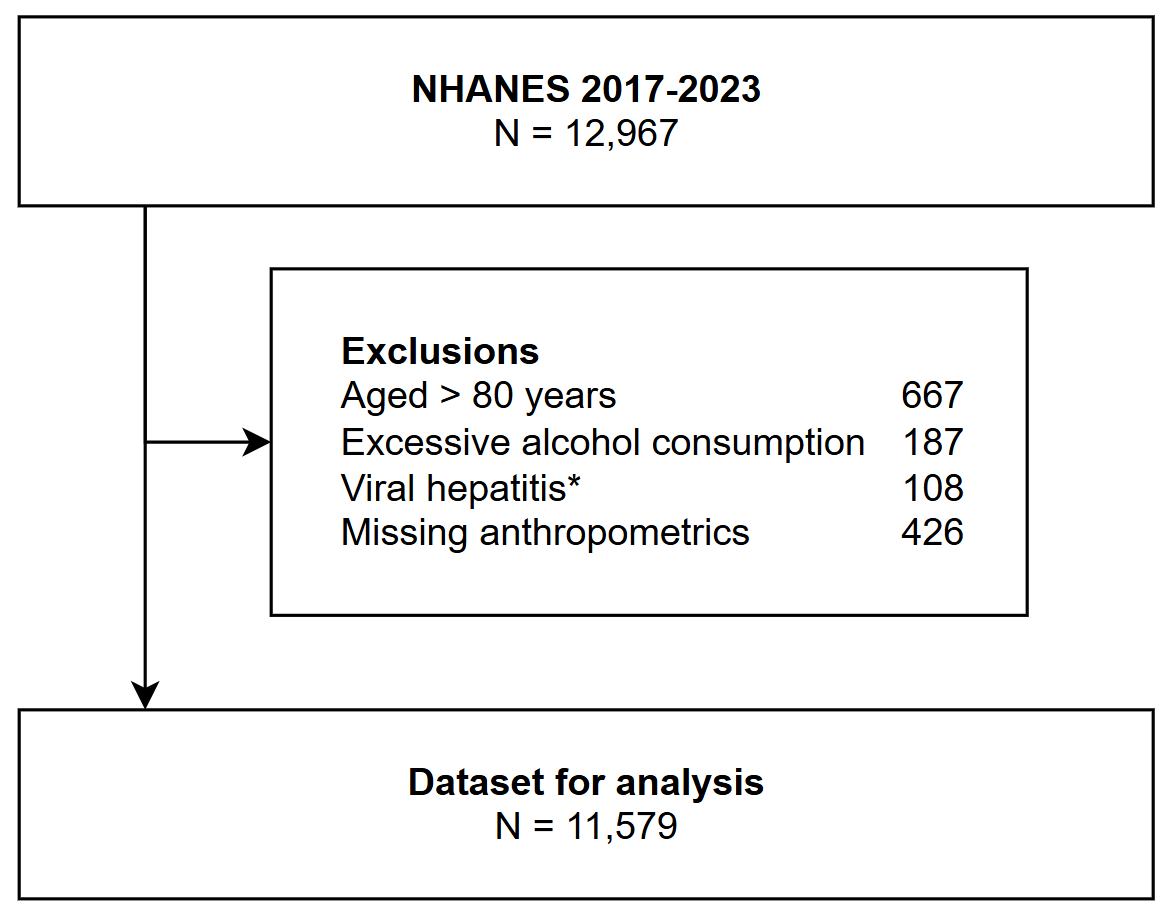
**Flowchart of participants with data on liver health for NHANES 2017-2023. * Only based on NHANES 2017-2020. Excessive alcohol consumption was defined as > 60 grams/day.

**Supplementary Figure 2: AUC per body composition marker for the detection of steatosis, at-risk MASH and increased liver stiffness in individuals with diabetes (A) and with metabolic dysfunction (B)**

**A**


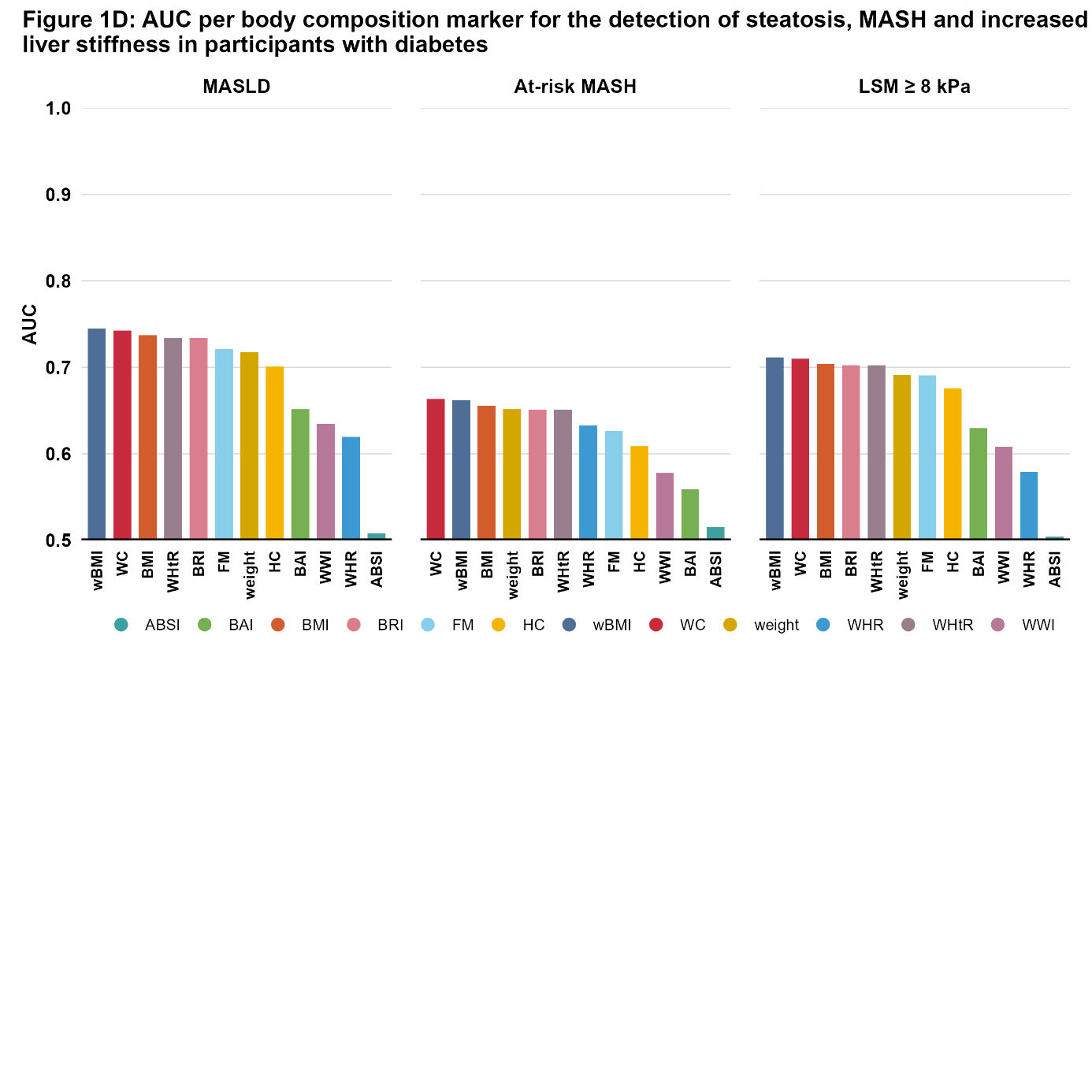


*****

*****

*****

*****

*****

*****

*****

*****

*****

*****

*****

**B**


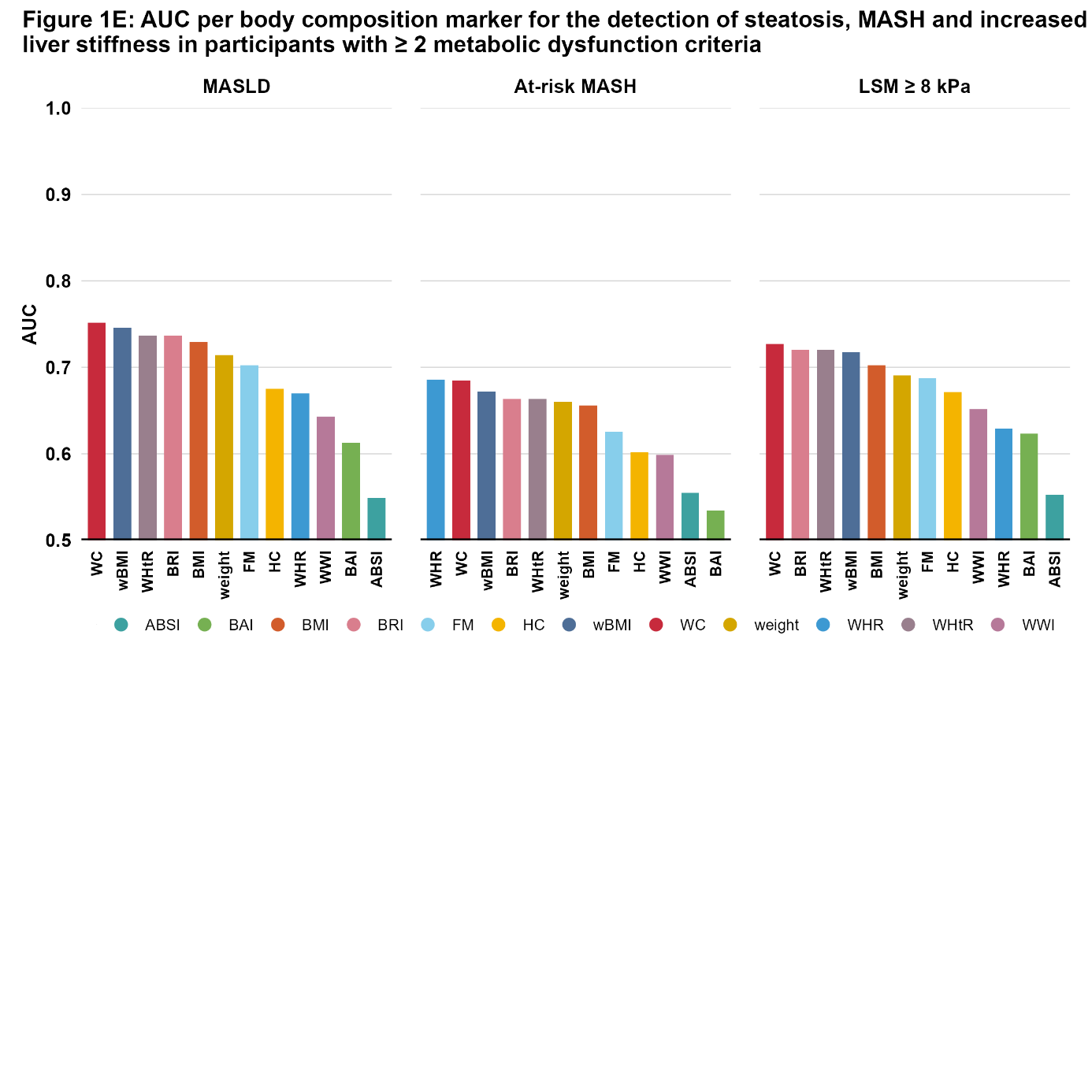


*****

*****

*****

*****

*****

*****

*****

*****

In individuals with diabetes, MASLD was present in 1420/2118, at-risk MASH in 167/1153 and LSM ≥ 8 kPa in 427/1967 participants. In individuals with at least two metabolic dysfunction criteria, MASLD was present in 4124/7753, at-risk MASH in 365/4229 and LSM ≥ 8 kPa in 981/7490 participants. AUC levels were compared among the body composition parameters using the DeLong test. * Indicates no significant difference with best performing test.

Abbreviations: ABSI, a body shape index; BAI, body adiposity index; BMI, body mass index; BRI, body roundness index; FM, fat mass; HC, hip circumference; LSM, liver stiffness measurement; MASLD, metabolic dysfunction associated steatotic liver disease; MASH, metabolic dysfunction associated steatohepatitis; BMI, waist adjusted BMI; WC, waist circumference; WHR, Waist Hip ratio; WHtR, Waist Height Ratio; WWI, weight adjusted waist index.
